# Dual Transcriptomic Analyses Unveil Host–Pathogen Interactions Between *Salmonella enterica* Serovar Enteritidis and Laying Ducks (*Anas platyrhynchos*)

**DOI:** 10.3389/fmicb.2021.705712

**Published:** 2021-08-05

**Authors:** Yu Zhang, Lina Song, Lie Hou, Zhengfeng Cao, Wanwipa Vongsangnak, Guoqiang Zhu, Qi Xu, Guohong Chen

**Affiliations:** ^1^Joint International Research Laboratory of Agriculture and Agri-Product Safety, The Ministry of Education, Yangzhou University, Yangzhou, China; ^2^College of Animal Science and Technology, Yangzhou University, Yangzhou, China; ^3^Department of Zoology, Faculty of Science, Kasetsart University, Bangkok, Thailand

**Keywords:** transcriptomics, dual RNA-seq, host–pathogen interactions, *Salmonella* enteritidis, duck

## Abstract

*Salmonella* enteritidis (SE) is a pathogen that can readily infect ovarian tissues and colonize the granulosa cell layer such that it can be transmitted via eggs from infected poultry to humans in whom it can cause food poisoning. Ducks are an important egg-laying species that are susceptible to SE infection, yet the host–pathogen interactions between SE and ducks have not been thoroughly studied to date. Herein, we performed dual RNA-sequencing analyses of these two organisms in a time-resolved infection model of duck granulosa cells (dGCs) by SE. In total, 10,510 genes were significantly differentially expressed in host dGCs, and 265 genes were differentially expressed in SE over the course of infection. These differentially expressed genes (DEGs) of dGCs were enriched in the cytokine–cytokine receptor interaction pathway via KEGG analyses, and the DEGs in SE were enriched in the two-component system, bacterial secretion system, and metabolism of pathogen factors pathways as determined. A subsequent weighted gene co-expression network analysis revealed that the cytokine–cytokine receptor interaction pathway is mostly enriched at 6 h post-infection (hpi). Moreover, a number of pathogenic factors identified in the pathogen–host interaction database (PHI-base) are upregulated in SE, including genes encoding the pathogenicity island/component, type III secretion, and regulators of systemic infection. Furthermore, an intracellular network associated with the regulation of SE infection in ducks was constructed, and 16 cytokine response-related dGCs DEGs (including *IL15*, *CD40*, and *CCR7*) and 17 pathogenesis-related factors (including *sseL*, *ompR*, and *fliC*) were identified, respectively. Overall, these results not only offer new insights into the mechanisms underlying host–pathogen interactions between SE and ducks, but they may also aid in the selection of potential targets for antimicrobial drug development.

## Introduction

Salmonellosis is an important foodborne illness that primarily occurs as a consequence of consuming eggs contaminated with *Salmonella* enteritidis (SE) (Gantois et al., 2009; Pijnacker et al., 2019) with more than 75% of SE outbreaks being linked to eggs or products derived therefrom (Braden, 2006). For example, an egg-linked international outbreak of SE infection has been occurring in the European Union for many years, posing a significant risk to consumer health and socioeconomic outcomes (Pijnacker et al., 2019). Although efforts to control SE outbreaks by vaccination and related interventional strategies to date have been made, these approaches have not completely prevented SE prevalence amid poultry flocks. Therefore, better strategies need to be further explored to control SE infection.

During SE infection, both the SE and the host mobilize all available resources to win the life-and-death struggle (Aprianto et al., 2016). One the one hand, SE encodes a number of pathogenic factors, such as PAMPs, including components of the bacterial cell wall–peptidoglycans, lipopolysaccharide, flagellin, and secretion system effectors to attack host innate immune (LaRock et al., 2015). Moreover, SE manipulates the cellular mechanisms of host organisms via pathogen–host interactions (PHIs) in order to take advantage of the capabilities of host cells, leading to infections; subsequently, transovarial transmission to offspring results in the spread of SE through the production of contaminated eggs and fecal matter (Gantois et al., 2009; Jennings et al., 2017). On the other hand, the host response to infection depends on innate immunity in which intrinsic mechanisms are responsible for recognizing and responding to SE challenge. The *NFKBIA*, *IL1B*, *IL8*, and *CCL4* genes were consistently induced after endotoxin treatment in chicken (Ciraci et al., 2010). Besides this, the *AVD*, *EXFABP*, *IRG1*, *AH221*, *TRAP6*, *SAA*, and all immunoglobulin genes also played important roles in the course of SE infection in chickens (Matulova et al., 2012). These findings only revealed the mechanism of *Salmonella* in the process of infection from host or pathogen. However, pathogen infection is a complex process involving the interaction between pathogen and host. Understanding of the host–SE interactions may reveal the underlying mechanism of SE infection, which will lay the theoretical foundation for better strategies to control the SE pathogen.

Ducks, especially Shaoxing and Pekin ducks, are an economically important poultry species, which are susceptible to SE infection (Yan et al., 2008; Cha et al., 2013; Zhang et al., 2019a). Notably, SE has recently grown to be the most common *Salmonella* serotype to be isolated from laying ducks throughout the world, and its prevalence in countries such as China and the United Kingdom have led to major economic losses while threatening human health and contaminating water (Martelli et al., 2016; Yang et al., 2019; Wang et al., 2020). SE is known to readily infect ovarian tissues and to colonize the granulosa cell layer in poultry (Thiagarajan et al., 1994). However, the PHIs between SE and duck granulosa cells (dGCs) have not been effectively defined. As such, we herein generated a time-resolved model of the SE infection of dGCs to simultaneously assess dynamic changes in gene expression in both duck and SE cells via a dual RNA-seq approach, enabling us to characterize the interactions between these two species and to identify key duck immune genes and SE virulence genes related to these responses.

## Materials and Methods

### Ethics Statement

The Institutional Animal Care and Use Committee of Yangzhou University (Jiangsu, China) approved all animal studies conducted herein, which were consistent with experimental animal use guidelines and regulations. All ducks were housed in a standard facility that met the requirements outlined in the Laboratory Animal Requirements of Environment and Housing Facilities (GB 14925-2001) publication, and all protocols were consistent with the Jiangsu Administration Rules for Laboratory Animal Use.

### Experimental Model Animals and Bacterial Strains

Healthy Shaoxing ducks (*Anas platyrhynchos*, Chinese native breed, 26 weeks old) that were free of *Salmonella* were obtained from the National Waterfowl Conservation Farm (Taizhou, China). SE (No. MY1, phage type 4) was isolated from ducks and maintained by the Key Laboratory of Animal Disease and Human Health of Sichuan Province (Deng et al., 2008). The National Center for Medical Culture Collection (Beijing, China) established the phage type and serotype of SE isolates.

### dGCs Isolation and SE Infection

Duck granulosa cells were isolated and cultured as in prior studies (Gilbert et al., 1977; Zhang et al., 2019b). Briefly, 10–15 adult prehierarchical follicles were collected from egg-laying ducks under sterile conditions. Yolks and vitelline membranes were removed by rinsing these follicles with Ca^2+^- and Mg^2+^-free PBS, after which tissues were minced into 1–2 mm^3^ pieces and digested with type II collagenase (1 mg/mL; Sigma, St. Louis, MO, United States) for 5 min at 37°C. Samples were then passed through a 200-μm nylon filter, centrifuged twice for 5 min at 67 × *g*, washed with M199 media to remove the remaining collagenase and cell debris, followed by resuspension in 3 mL of 50% Percoll, centrifugation at 421 × *g* for 15 min, and cell layer isolation. The dGCs were then suspended in M199 media (5% fetal calf serum, 2 mmol/L L-glutamine, 5 μg/mL transferrin, 10 μg/mL insulin, 1.75 mM HEPES [4-(2-hydroxyerhyl) piperazine-1-erhanesulfonic acid]) and counted with 0.1% trypan blue being added to assess viability. Only samples with >90% cell survival were used for subsequent experiments. Cells were allowed to adhere in tissue culture flasks for 24 h prior to experimental utilization, after which purity was initially determined by indirect immunofluorescence assay (IFA) to detect follicle granulosa cell-specific receptor, follicle-stimulating hormone receptor (FSHR) expression using anti-FSHR antibodies (1:500, Proteintech, Rosemont, IL, United States, L594-22665).

### Invasion Assays

Invasion assays were conducted as in prior reports (Jouve et al., 1997; Lane and Mobley, 2007). Briefly, bacteria were cultured in LB broth to an OD_600_ of 2.0 (at mid logarithmic phase) at 37°C. dGCs were added to 96-well plates (1 × 10^5^/well) for 24 h, after which they were washed thrice with PBS (pH 7.2) prior to the addition to each well of 100 μL of SE in Dulbecco’s modified Eagle medium (DMEM) at a multiplicity of infection (MOI) of 10 with DMEM being added to control wells. Plates were then cultured 37°C for 1 h, after which PBS supplemented with 50 μg/mL gentamicin was added to kill any non-invasive bacteria. Cells were then incubated for an additional 1 h period and then lysed by 0.5% Triton X-100 (Solarbio, Beijing, China) for 30 min before vigorous pipetting. Samples were removed, diluted, and plated on LB agar to determine the number of colony forming units (CFU) per monolayer. Samples were assessed in triplicate.

*Salmonella* enteritidis infection was confirmed via an IFA approach as detailed previously (Zhang et al., 2019c). Briefly, medium was removed, and cells were washed three times with 0.01 M PBS, fixed with 4% paraformaldehyde, permeabilized with 0.1% Triton X-100, and blocked for 1 h with 10% FBS in PBS. After a 2-h incubation with an anti-*Salmonella* antibody (1:2000, Abcam, Cambridge, MA, United States, ab69253), cells were washed thrice with PBS, stained with DAPI (0.2 mg/mL) for 15 min at 37°C, and imaged via fluorescence microscopy (Leica, Wetzlar, Germany).

### RNA-Sequencing

TRIzol (Invitrogen, Carlsbad, CA, United States) was used based on provided directions to extract RNA from 0, 3, 6, and 9 hpi groups (*n* = 3, respectively) with 1% agarose gel electrophoresis being used to assess RNA quality and contamination. A NanoPhotometer spectrophotometer (IMPLEN, Westlake Village, CA, United States) was also used to assess RNA purity, and a Qubit RNA Assay Kit and a Qubit 2.0 Fluorometer (Life Technologies, Carlsbad, CA, United States) were used to measure RNA concentrations. An RNA Nano 6000 Assay Kit and a Bioanalyzer 2100 system (Agilent Technologies, Santa Clara, CA, United States) were then used to assess RNA integrity. Next, a strand-specific library was constructed followed by the previous method (Parkhomchuk et al., 2009; Borodina et al., 2011). Briefly, oligo (dT) beads were used to enrich for eukaryotic RNA in each sample first, then prokaryotic mRNA was enriched via the removal of rRNA with a Ribo-Zero Magnetic Kit (Epicentre) and Quick-RNA^TM^ Fungal/Bacterial Microprep (Zymo Research). After enrichment, fragmentation buffer was used to fragment mRNA into short segments, after which first-strand DNA synthesis was conducted using random hexamer primers. Second-strand cDNA synthesis was then performed with DNA polymerase I, RNase H, dNTPs, and an appropriate buffer. A QiaQuick PCR extraction kit was then used for cDNA isolation, followed by end repair, polyadenylation, and Illumina sequencing adapter ligation. Ligation product size selection was then performed via agarose gel electrophoresis, followed by PCR amplification and sequencing runs of paired-end 150 bp with an Illumina HiSeq 2500 instrument at Major BioTech Co., Ltd. (Shanghai, China).

Raw reads were trimmed and subjected to quality control using Sickle^1^ and SeqPrep^2^ with default settings. After cleaning, data were mapped to the duck [NCBI: IASCAAS_PekingDuck_PBH1.5 (GCF_003850225.1)] and SE (NC_011294.1) reference genomes using TopHat2 and Bowtie2, respectively (Trapnell et al., 2010; Langmead and Salzberg, 2012). The “MarkDuplicate” tools of Picard^3^ were used removed the reads that mapped to both organisms. Relationships among samples were assessed with the gmodels R package^4^.

### Differentially Expressed Gene (DEG) Analyses

The gene abundances were quantified by software RSEM, and the gene expression level was normalized by using the fragments per kilobase of transcript per million mapped reads method (Li and Dewey, 2011). Differentially expressed genes (DEGs) among samples were identified with the edgeR package (Robinson et al., 2010). DEGs were defined as those genes with a | log2FC| ≥ 2 and a false discovery rate (FDR) < 0.05. After identification, Blast2GO (Conesa and Götz, 2008) was utilized for the GO annotation of these genes (Conesa et al., 2005), and the KEGG program was utilized for KEGG pathway enrichment analyses (Kanehisa and Goto, 2000). The weighted gene co-expression network analysis (WGCNA) R package was used to conduct a WGCNA of gene expression patterns across samples (Langfelder and Horvath, 2008). Following pair-wise correlation matrix calculation, an adjacency matrix was calculated by raising the correlation matrix based on a power value (β) that had been selected based upon scale-free topology criteria (scale-free topology criteria *R*^2^ ≥ 0.85). Topological overlap was then assessed. A cluster tree branch gene co-expression module was constructed based upon average linkage hierarchical clustering. Module eigengenes were statistically classified (dynamicTreeCut algorithm, parameters deepSplit = 2, minModulesize = 30) (Langfelder et al., 2008), after which very similar modules were merged, leading to the identification of key host genes associated with responses to SE infection at different time points. Clusterprofiler and DAVID were used for KEGG and GO enrichment analysis, respectively (Huang et al., 2009; Yu et al., 2012).

### Interaction Network Construction and Prediction of the Regulation of Pathogenicity-Associated Genes

Genes involved in PHIs were identified by using BLASTp to compare potentially relevant genes against the PHI-base version 4.8 database (September 2019) (Urban et al., 2020). Network-based inferences necessitating a gene expression profile is centralized and standardized using the min-max normalization method (Zhu et al., 2019). Gene expression pattern analysis is used to cluster genes of similar expression patterns for multiple samples. To examine the expression pattern of DEGs, the expression data of each sample (in the order of treatment) were normalized to 0, log2 (v1/v0), log2 (v2/v0), and then clustered by Short Time-series Expression Miner software (STEM) (Ernst and Bar-Joseph, 2006). Host dGCs DEGs (euk-DEGs) and prokaryotic SE-associated DEGs (pro-DEGs) were then selected to predict associations between key virulence genes and host genes via an expression correlation analysis. Interaction networks of euk-DEGs and corresponding pro-DEGs were constructed using Cytoscape (Shannon et al., 2003). To improve network visibility, the edges (connections) were filtered to show only those with a degree cutoff = 2, a node score cutoff = 0.2, k-core = 2, and max depth = 100. The two significant networks were then calculated with the MCODE plug-in (Bader and Hogue, 2003).

### qPCR-Based Result Validation

In order to validate RNA-seq results via qPCR, 12 duck genes (*IL15*, *CCR7*, *IL18R1*, *CD40*, *IL23R*, *IL20RA*, *TNFRSF8*, *TNFRSF13B*, *TNFRSF4*, *CSF3R*, *IL2RG*, *TNFRSF18*) and 17 SE virulence-associated genes (*sseL*, *sseE*, *invE*, *ssaM*, *ssaN*, *ssaP*, *spiC*, *slyA*, *spaR*, *aroA*, *sipB*, *prgH*, *hilD*, *ompR*, *fruR*, *fliC*, *sipA*) were selected for analysis with *GAPDH* and *gyrA* being used for the respective normalization of duck and SE transcript expression levels. Primers were designed with the Primer 5 software (Premier, Canada) based upon sequencing data (Table 1), and were synthesized by Sangon Biotech (Shanghai, China). TRIzol (Takara, Dalian, China) was used based on provided directions to extract RNA from prepared samples. All qPCR reactions were conducted with an ABI 7500 instrument (Applied Biosystems, Foster City, CA, United States) with individual reactions being composed of 10 μL 2 × SYBR Premix Ex Taq II (Takara), 0.4 μL of each primer (10 μM), 0.4 μL 50 × ROX Reference Dye II (Takara), 2 μL cDNA, and ddH_2_O to a final volume of 20 μL. Thermocycler settings were 95°C for 30 s, 40 cycles of 95°C for 5 s, 60°C for 34 s. The thermal program for RT-qPCR was 98°C for 30 s, followed by 40 cycles of 98°C for 10 s and 60°C for 30 s. All samples were conducted with three technical replications. The 2^–ΔΔCT^ method (Kenneth and Thomas, 2002) was used to assess relative gene expression levels.

**TABLE 1:** Primers of genes used in RT-qPCR.

Primer	Sequences (5′-3′)	Accession number	Size (bp)
*IL15*	F: TGTTGTTCCCACCCTC R: AGCATCTTGCCTCCTG	XM_013109782.3	150
*CCR7*	F: CTGGCATCAAAGTATCC R: TGACGCTGTTGTAGGG	XM_005030336.5	186
*IL18R1*	F: TCATTGCCGTGGTTATCG R: AAGCAGGCCAGCGTGTA	XM_038173244.1	154
*CD40*	F: CAGGGCTTTGGGTTTG R: TAGCACGGCTCGGTTT	XM_027442951.2	110
*IL23R*	F: TCACCTACATCCAAGAAGACA R: CCCATTCCCAGGTAAACTC	XM_038183055.1	181
*IL20RA*	F: CAAACCTGGTATGGCACT R: GCACTTCTTTTGGAGGC	XM_027454461.2	207
*TNFRSF8*	F: ACAGCCATCGCTCACCT R: GCTTTTGGGATTCTTCGT	XM_013102263.4	188
*TNFRSF13B*	F: GCATCGGAGGGATAAGG R: TGGTCGGTGCAGTTGTT	XM_013099438.3	208
*TNFRSF4*	F: ATTTGGTGGGGACGGT R: GCGACTTCTCATCCTTTCA	XM_013102273.4	189
*CSF3R*	F: GAAGAGCATCAGCAAGGC R: TGCTTCACCCTCCCATT	XM_038167306.1	360
*IL2RG*	F: CAGGACCTCGTGAAACC R: GCTGGGGAAGGAGAAGAT	XM_027464860.2	192
*TNFRSF18*	F: CAGAAATCAAGGACACGG R: AGTCAATAATGCCACGCT	XM_013103783.4	129
*GAPDH*	F: TGCTAAGCGTGTCATCATCT R: AGTGGTCATAAGACCCTCCA	XM_038180584.1	60
*sseL*	F: GAATCAGCCCAATAGGATAG R: ACCAGTTCGCTCAGACAGA	AM933172.1	153
*sseE*	F: AGCCATGCTACGCAGGAAA R: GATGCTCGGCGGATAAAACT	AM933172.1	101
*invE*	F: TCCCGGCAGACATCTCAT R: AAGCCTCATCCTCCAGCAC	AM933172.1	226
*ssaM*	F: TCTGGCGGCAAGGACAATA R: GAAAGAGGTGGAGAACAAC	AM933172.1	205
*ssaN*	F: CTTTGGTCGTCCCCTTGA R: TACCCACTCGTTGCCCTTC	AM933172.1	167
*ssaP*	F: GTTGAGGGAAGTCTTGGGTT R: ATGGGTAATGCCTGGTGC	AM933172.1	104
*spiC*	F: ATGCTGGCAGTTTTAAAAGGCATTC R: CATAGGCAAGACAAGGCT	AM933172.1	181
*slyA*	F: CATCGCCTCAAGCCTCT R: CGCTTTCTCGGTCAGTT	AM933172.1	225
*spaR*	F: CTCCGCAAATGAACGCTT R: CCTCGCTCGTAAAACCAACT	AM933172.1	154
*aroA*	F: CGAACCACCACTACCAACAA R: GAAAATAGGACGCTGACGAG	AM933172.1	102
*sipB*	F: TGGAAGGATTAGGCGTCG R: CCTGGGTAAAGAGTTTGCTG	AM933172.1	234
*prgH*	F: GCTGTGAGTTTCCATTGCTG R: CCGACCTGTATTGGCGTATT	AM933172.1	241
*hilD*	F: CAGACTCAGCAGGTTACCATCA R: GTCGTTGCGTCGGTATCTC	AM933172.1	217
*ompR*	F: GATTCTGGTGGTTGATGACG R: GATGGAAAGATTCACGGGTC	AM933172.1	134
*fruR*	F: AGCATCCCTTCTATCAGCG R: GCCCAAATAAAGCACCGT	AM933172.1	170
*fliC*	F: ATTGAGCGTCTGTCCTCTGG R: GATTTCATTCAGCGCACCTT	AM933172.1	170
*sipA*	F: CCATTCGACTAACAGCAGCA R: CGGTCGTACCGGCTTTATTA	AM933172.1	449
*gyrA*	F: GCATGACTTCGTCAGAACCA R: GGTCTATCAGTTGCCGGAAG	MG995181.1	278

### Statistical Analyses

Data are means ± standard deviation (SD), and experiments were repeated at least three times. Data were analyzed with SPSS 26.0 (Chicago, IL, United States) via one-way ANOVAs with Dunnett’s test, with *p* < 0.05 as the significance threshold.

### Data Access

RNA-seq data from the present study have been deposited in the Short Read Archive of the National Center for Biotechnology Information (NCBI) under the bio-project number PRJNA721551.

## Results

### Evaluation of dGC Invasion by SE

**FIGURE 1:**
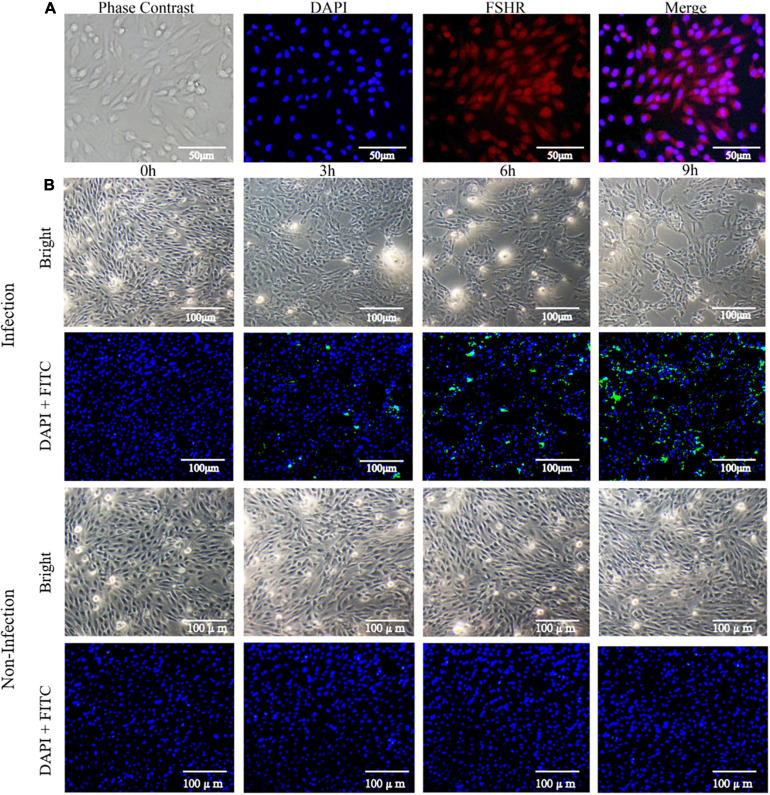
Isolation of dGCs and challenge with SE. **(A)** Isolation and identification of dGCs. Diagram of a dGC (400×), DAPI staining of cell nucleus, and fluorescent image of FSHR. **(B)** The changes of morphology and indirect immunofluorescent staining and DAPI staining of dGCs infected at an MOI of 10 at different times post-infection. The row represents 0, 3, 6, and 9 hpi, respectively.

After isolation, dGCs exhibited a rounded or ovoid shape and were fully adherent to tissue culture flasks within 24 h of culture at which point they were spread out in a pebble-like manner. These cells reached 100% confluence within 3–5 days, at which time granular material was evident in the cytoplasm. DAPI was used to stain dGCs nuclei, and the cytoplasm of the FSHRs was stimulated to emit red fluorescence for detection by IFA (Figure 1A). After treatment with bacterial suspensions and gentamicin-containing growth media, changes in cell morphology and numbers of invasive SE were assessed at different time points post-infection. At 3 hpi, cells appeared swollen and rounded or irregular in shape with some evidence of vacuolation. At 6 hpi, infected cells were increasingly non-adherent with some blue or black particles, cellular deformation, and clumping in strip-like aggregates. At 9 hpi, some cells had broken down and dissolved with increases in the presence of dark blue particles within dGCs. The media also appeared increasingly flocculated at this time point due to cell exfoliation. IFA staining of the SE in these samples revealed that the degree of bacterial invasion increased significantly over time such that relatively few bacteria were detectable at 3 hpi, whereas these bacterial numbers had expanded significantly by 6 hpi and had invaded most cells by 9 hpi (Figure 1B). The intracellular CFU counts of SE at each time point are listed in Supplementary Table 1.

### Profiling of Gene Expression Patterns Associated With HPIs

**FIGURE 2:**
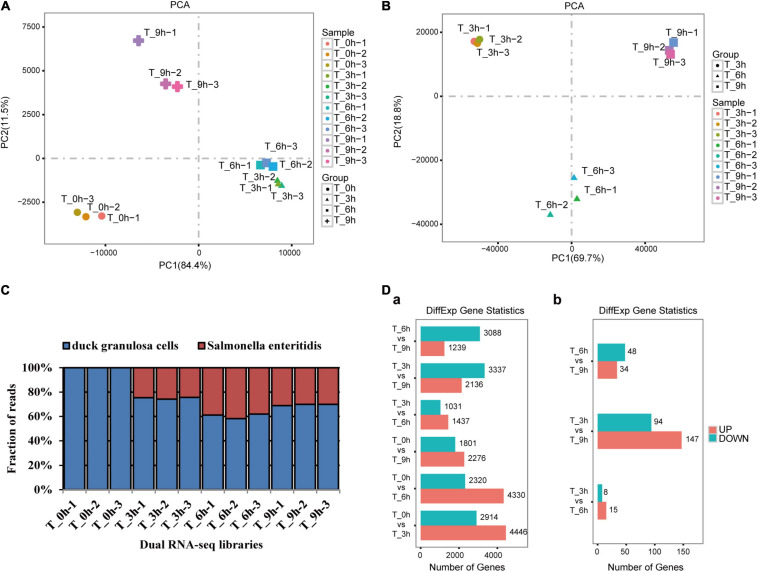
Dual RNA-seq generates high-quality data sets suitable for probing host–pathogen transcriptomes. Principal component analysis was performed separately for dGCs and SE libraries. Biological duplicates clustered closely to each other in host **(A)** and pathogen **(B)** libraries (see sample duplicates). Variability within samples was mainly dependent on the infection time points but not sequencing batch of the samples; 78.6 million reads per library: 76.2% of the reads aligned to the duck reference genome and 23.8% to the SE P125109 reference genomes **(C)**, the DEG statistics at different specific stage of dGCs (a) and SE (b) interaction **(D)**.

To examine dGCs and SE gene expression profiles over the course of this infection process, we conducted a dual RNA-seq time-course analysis of both dGCs and SE cells at 0, 3, 6, and 9 hpi (Supplementary Table 2). This RNA-seq approach yielded high-quality yields with an appropriately balanced base distribution and a reasonable N% (>10%) consistent with these data being of sufficient quality to permit downstream analyses (Supplementary Table 3). Average base error rate distributions in these sequencing reads were <0.1% and were, thus, considered acceptable. Principal component analysis clustering was then conducted to confirm sample distribution and biological reproducibility (Figures 2A,B). The gmodels R package was used to evaluate the relationships among samples (Supplementary Figure 1). On average, this analysis yielded 78.6 million reads per library with 76.2% of these reads aligning to the duck reference genome and 23.8% aligning to the SE P125109 reference genome (Supplementary Table 4 and Figure 2C). The edgeR package (Robinson et al., 2010) was used to assess gene expression profiles and to identify significant DEGs that met the defined criteria (FDR < 0.05 and | log2FC| > 1) at different time points in both dGCs and SE cells (Figure 2D). For further details regarding DEGs and virulence-associated genes between the 0, 3, 6, and 9 hpi time points and associated biological functions, see Supplementary Tables 5, 6.

### Enrichment Analysis of Pathways and GO Terms for DEGs in SE and dGCs

**FIGURE 3:**
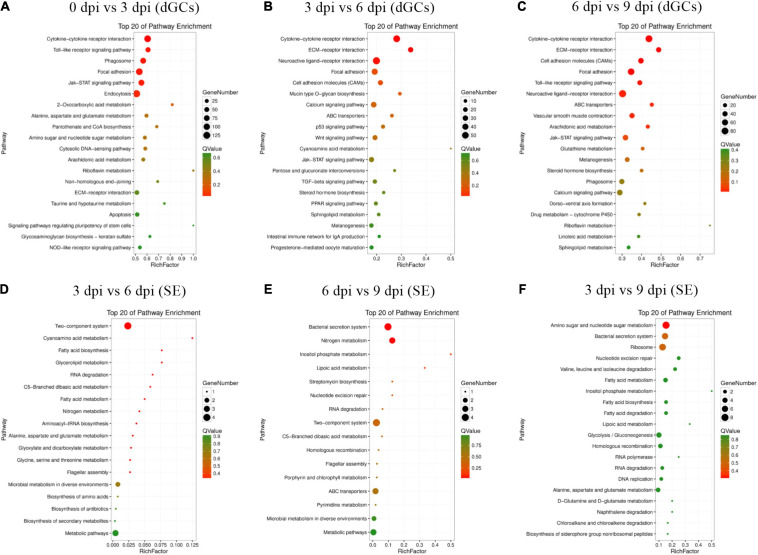
Top 20 enriched KEGG pathways for difference expression genes between the different stages of the SE infection. All DEGs in specific stage infection were analyzed by KEGG enrichment. Fold change > 2 and FDR < 0.01 was set as the cutoff values: 0 vs. 3 hpi **(A)**, 3 vs. 6 hpi **(B)**, 6 vs. 9 hpi **(C)** of dGCs in different contrasts, respectively. 3 vs. 6 hpi **(D)**, 6 vs. 9 hpi **(E)**, 3 vs. 9 hpi **(F)** of SE in different contrasts, respectively.

GO analyses of identified DEGs in dGCs and SE were conducted (Supplementary Tables 7, 8), revealing these genes to be associated with GO terms mainly enriched in host immune function, including *immune system process* (GO: 0002376), *regulation of cytokine production* (GO: 0001817), *positive regulation of biological process* (GO: 0001819), and *regulation of cellular metabolic process* (GO: 0031323). Bacterial DEGs were additionally associated with bacterial virulence–related protein secretion, such as *the metabolic process* (GO: 0008152), *peptide biosynthetic process* (GO: 0043043), and *regulation of gene expression* (GO: 0010468) GO terms (Supplementary Figure 2). In addition, the top 20 enriched KEGG pathways associated with genes that were differentially expressed during different stages of the SE infection process were assessed (Figure 3). These pathways included the cytokine–cytokine receptor interaction, Toll-like receptor signaling, JAK-STAT signaling, ECM-receptor interaction, and p53 signaling pathways, which play key roles in immune, signal transduction, and transcription-related processes. Statistical analyses suggest that the cytokine–cytokine receptor interaction pathway in dGCs was significantly altered during SE infection as were the two-component system and bacterial secretion system pathways in SE (Table 2). For further details regarding the DEGs associated with the cytokine–cytokine receptor interaction, two-component system, and bacterial secretion system pathways in this experimental system, see Table 3.

**TABLE 2:** Top 2 significantly changed pathways in different group contrasts and specific modules.

Class	Group/module	Term	Count	Percent (%)	*p*-Value	*Q*-Value
dGCs	0 vs. 3 hpi	Cytokine-cytokine receptor interaction Toll-like receptor signaling pathway	11253	5.882.78	9.34 × 10^–8^ 1.93 × 10^–4^	1.62 × 10^–5^ 1.34 × 10^–2^
	3 vs. 6 hpi	Cytokine-cytokine receptor interaction ECM-receptor interaction	5227	8.934.64	8.42 × 10^–9^ 7.64 × 10^–7^	1.17 × 10^–6^ 5.31 × 10^–5^
	6 vs. 9 hpi	Cytokine-cytokine receptor interaction ECM-receptor interaction	8139	7.973.84	1.85 × 10^–11^ 1.17 × 10^–7^	3.05 × 10^–9^ 9.68 × 10^–6^
dGCs	Blue (0 hpi)	Metabolic pathways Biosynthesis of amino acids	23721	28.402.51	2.20 × 10^–3^ 1.40 × 10^–3^	0.160.16
	Darkred (3 hpi)	Valine, leucine and isoleucine degradation Tryptophan metabolism	97	3.412.65	1.20 × 10^–3^ 7.05 × 10^–3^	0.160.26
	Black (6 hpi)	Cytokine-cytokine receptor interaction Wnt signaling pathway	4831	7.234.67	2.15 × 10^–5^ 1.22 × 10^–3^	3.6 × 10^–3^ 0.10
	Lightcyan (9 hpi)	Cell cycle DNA replication	3115	7.403.58	6.07 × 10^–8^ 2.71 × 10^–8^	4.13 × 10^–6^ 3.68 × 10^–6^
SE	3 vs. 6 hpi	Two-component system Cyanoamino acid metabolism	41	40.0010.00	1.88 × 10^–2^ 5.18 × 10^–2^	0.300.30
	6 vs. 9 hpi	Bacterial secretion system Nitrogen metabolism	43	19.0514.29	1.99 × 10^–3^ 3.87 × 10^–3^	0.030.03
	3 vs. 9 hpi	Amino sugar and nucleotide sugar metabolism Bacterial secretion system	86	10.007.50	4.83 × 10^–3^ 1.84 × 10^–2^	0.310.50

*KEGG pathway analysis was performed with DAVID.*

*p-value < 0.05 was considered to be statistically significant.*

**TABLE 3:** The significantly changed pathways and enrichment different expression genes of host and bacteria during SE infection.

Class	DEGs	Function
dGCs	*IL22RA1*, *IL23R*, *TNFSF4*, *IL7*, *IL18*, *TNFRSF13B*, *TNFRSF8*, *CCL19*, *IL15*, *TNFRSF4*, *TNFSF8*, *TNFRSF1B*, *CCR7*, *TNFSF11*, *IL20RA*, *TNFRSF18*, *CSF3R*, *TNFRSF19*, *IL2RG*, *NGFR*, *IL13RA1*, *IL18R1*, *IL1R2*, *IL6*, *IL21R*, *TNFSF15*, *CD40*, *IL7R*, *AMH*, *TNFRSF9*, *TNFRSF11B*, *TNFSF10*, *CCL20*, *CXCL14*	Cytokine–cytokine receptor interaction
SE	*fliC*, *ttrB*, *pagC*, *ompR*, *pagO*, *narG*, *ssrA*, *ssrB*, *pagC*, *htrA*, *ompC*, *pids*	Two-component system
	*ssaU*, *yscR*, *ssaV*, *ssaJ*, *ssaC*, *tolC*, *BPSS1504*, *ssaL*, *ssaK*, *sseD*, *sseC*, *ssaI*	Bacterial secretion system

### Identification of Key Genes Associated With dGC Responses to Infection and SE Pathogenicity

**FIGURE 4:**
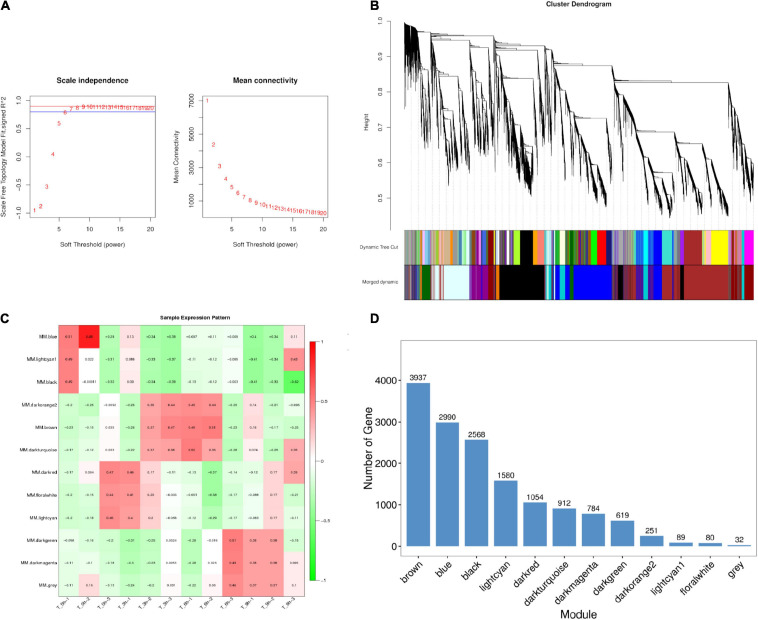
Identification and visualization of stage-specific modules based on WGCNA. **(A)** Analysis of the scale-free fit index for soft-thresholding powers (β) and the mean connectivity for soft-thresholding powers. **(B)** Clustering dendrogram of genes showing module membership in colors. **(C)** Heat map of correlations between module and specific infection stage. The colors ranging from green through white to red indicate low through intermediate to high correlations, respectively. MM, the first principal component of the standardized expression profiles of a given module (absolute correlation greater than 0.4 and *p*-value less than 0.01) are indicated significant. **(D)** Distribution of average gene significance in the modules associated with the stage-specific modules.

A WGCNA analysis was conducted to assess relationships between particular genes during a given stage of the infection process, grouping similarly expressed genes into modules via average linkage clustering (based on the weighted correlation coefficients of genes, genes are classified according to their expression patterns, and genes with similar patterns are grouped into one module). Herein, a power value of β = 9 (scale-free *R*^2^ = 0.85) was utilized for oft-thresholding to ensure a scale-free network (Figure 4A), leading to the identification of 12 modules (Figure 4B). Module connectivity was assessed based upon Pearson’s correlation coefficient values (cor.geneModuleMembership > 0.8) with clinical trait relationships similarly being measured based upon absolute Pearson’s correlation coefficient values (cor.geneTraitSignificance > 0.4). As the identification of modules significantly associated with clinical features was considered to be of biological importance, module–feature relationships were assessed at different pathological stages, revealing that the most closely associated modules at the 0, 3, 6, and 9 hpi time points were the blue, dark red, black, and light cyan modules, respectively (Figures 4C,D and Supplementary Table 9). These four modules were, thus, selected as modules of interest with clinical features worthy of examination in subsequent analyses. Genes in these significant modules at the 0, 3, 6, and 9 hpi time points were associated with metabolic pathways, the MAPK and JAK-STAT signaling pathways, the cytokine–cytokine receptor interaction pathway, and the cell cycle and p53 signaling pathways, respectively (Table 2 and Supplementary Figure 3). The key genes in the cytokine–cytokine receptor interaction pathway within the black modules are additionally listed in Table 4. To further understand SE pathogen-related factor expression over the course of the infection process, identified genes were subjected to a BLAST analysis and annotation of high-abundance genes (expression > 10) using the PHI database with identified pathogenicity-associated factors being listed in Table 4.

**TABLE 4:** The key genes and pathologic factors based on pattern expression of specific module and PHI database.

Class	Key genes/pathologic factors	Function
dGCs	*TNFRSF1A*, *CD40*, *CCR5*, *CCR7*, *CXCR4*, *EGF*, *EGFR*, *PDGFRA*, *IL1R1*, *TGFBR2*, *ACVR2A*, *TNFRSF10A_B*, *IL1RAP*, *CSF2RB*, *CSF3R*, *IL23R*, *CSF2RA*, *IL2RB*, *IL2RG*, *CSF1R*, *IL10RA*, *IL20RA*, *IL22RA1*, *IL22RA2*, *IFNLR1*, *TNFRSF1B*, *TNFRSF4*, *TNFRSF6B*, *TNFRSF8*, *TNFRSF13B*, *TNFRSF18*, *TNFRSF21*, *IL18R1*, *IL12B*, *IL15*, *IL19*, *TNFSF8*, *CCL19*, *CXCL13*, *CCL5*, *CCL4*, *CCL21*	Cytokine–cytokine receptor interaction
SE	*slyA*, *ssrB*, *hfq*, *csrA*, *smpB*, *ssrA*, *rpoE*, *himD*, *crp*, *hnr*, *envZ*, *ompR*, *fruR*, *hilD*, *hnr*, *ropS*	Regulators of systemic infection
	*sseL*, *sseB*, *spiC*, *ssaM*, *ssaV*, *purA*, *aroA*, *ttrB*, *fliC*, *BPSS1504*	Pathogenicity island/component
	*ssaJ*, *sseC*, *ssaC*, *ssaL*, *ssaK*, *ssaT*, *sseE*, *ssaE*, *ssaS*, *ssaU*, *ssaN*, *ssaQ*, *sseD*, *ssaP*, *ssaI*, *sseF*, *sscB*, *lon*, *invE*, *sifA*, *spaR*, *sipB*, *prgK*, *prgH*, *sipA*, *yscR*	Type III secretion

### Co-expression Analysis of DEGs in SE and dGCs

**FIGURE 5:**
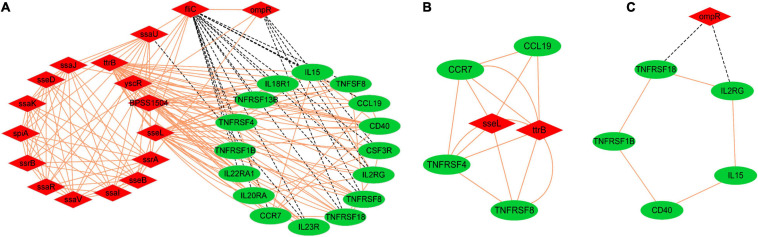
The interaction relationships among the host DEGs of cytokine–cytokine receptor interaction pathways and the pathogenic expression genes of SE. Sixteen host genes interaction with 17 pathogenicity-associated factors during infection **(A)**, Degree cutoff = 2, node score cutoff = 0.2, k-core = 2, and max. depth = 100 as the criteria two significant modules were selected by using plug-in MCODE, Score = 4.8 **(B)**, Score = 2.4 **(C)**, *p*-value < 0.05, *P*_*x,y*_ > 0.6. Red and green color represent bacteria and host, respectively, red solid lines represent positive relationship, and black dashed lines represent negative relationship.

Fuzzy c-means clustering (Bezdek, 1992) and expression correlation analyses were next used to evaluate the expression matrices of DEGs from dGCs and SE cells (Supplementary Figures 4,5). This led to the identification of 16 host genes associated with the cytokine–cytokine receptor interaction pathway that exhibited high fold-change expression patterns during the infection process as well as 17 pathogenicity-associated factors that were primarily associated with functions including regulators of systemic infection, pathogenicity island/component, and type III secretion system components (<0.05, *P*_*x,y*_ > 0.6) (Figure 5). In Figure 5, host and pathogen DEGs are, respectively, represented by circles and inverted triangles. The expression levels of most host cytokines were positively correlated with the expression of bacterial virulence factors, particularly the type III secretion-related proteins; bacterial virulence factors *ompR*, *fliC*, and *ssaU* were the negative regulators to the expression of host response genes; and *sseL*, *ttrB*, and *ompR* interact throughout the higher cooperative diversity in the regulatory network.

### qPCR-Based Validation of Hub Genes and Pathogenicity-Associated Factors

**FIGURE 6:**
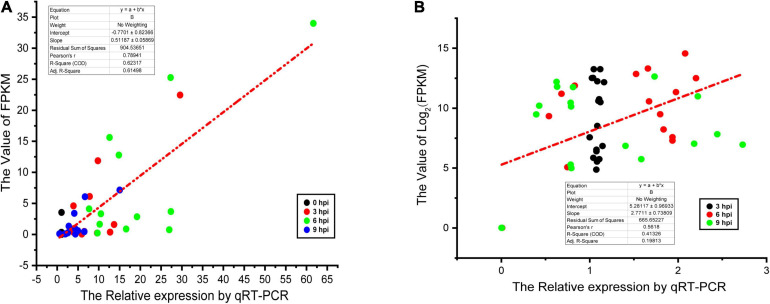
Linear regression fitted for Log_2_ fold change of selected genes determined via qPCR and RNA-seq. The selected genes in each comparison were used for linear regression analysis. Log_2_FC in RNA-seq equals 2^– ΔΔCt^ in qPCR for each comparison. **(A)** The results of dGCs. **(B)** The results of SE.

Last, a qPCR approach was used to validate the DEGs identified in the above RNA-seq analysis. In total, 12 hub genes (*IL15*, *CCR7*, *IL18R1*, *CD40*, *IL23R*, *IL20RA*, *TNFRSF8*, *TNFRSF13B*, *TNFRSF4*, *CSF3R*, *IL2RG*, and *TNFRSF18*) and 17 pathogenicity-associated factors (*sseL*, *sseE*, *invE*, *ssaM*, *ssaN*, *ssaP*, *spiC*, *slyA*, *spaR*, *aroA*, *sipB*, *prgH*, *hilD*, *ompR*, *fruR*, *fliC*, and *sipA*) were selected for validation. Subsequent qPCR results appeared similar to the outcomes of the dual RNA-seq analysis, thus confirming the validity of these RNA-Seq results (Figure 6).

## Discussion

*Salmonella* enteritidis is a zoonotic pathogen that can readily colonized the granulosa cell layer of ovarian tissues (Gantois et al., 2009). Clarifying HPIs will help reveal the mechanism for regulating the virulence of pathogenic bacteria. In this study, the 10,510 DEGs in host dGCs and 265 in SE were identified during SE infection, and 16 cytokine response-related dGCs DEGs (including *IL15*, *CD40*, and *CCR7*) and 17 pathogenesis-related factors (including *sseL*, *ompR*, and *fliC*) were identified. These results were similar to those of chicken granulosa cells or duck ovary tissues challenged by SE (Tsai et al., 2010; Babu et al., 2016; Zhang et al., 2019a). Other genes, including *IAP1*, *CD28*, *TGF*β*2-4*, *Gal11-13*, *TRAIL*, *PSAP*, *CASP1*, *AVD*, *EXFABP*, *IRG1*, and *AH221*, et al., played an important role of anti-SE immune regulation in cecum and spleen (Calenge and Beaumont, 2012; Matulova et al., 2012, 2013). Together, these data suggest that the immune response of host immune genes were different in different tissues and organs during SE infection. *Salmonella*, as a pathogen of vertical transmission through the ovary, should establish an ovary-specific immune response during the infection process.

Furthermore, the cytokine–cytokine receptor interaction pathway, two-component system, and bacterial secretion system were enriched to involve HPIs. Results of the present study were not in agreement with the findings of Huang et al. (2016) and Wang et al. (2019), who demonstrate that the IgA production signaling pathway and TLR4-FOS/JUN pathway contributed to the protection of chicken from *Salmonella* invasion, respectively. The reason for these different pathways might be due to sequencing methods. In the present study, the dual transcriptomic analyses were performed to unveil HPIs. Westermann and Vogel (2018) prove that dual RNA sequencing is an effective strategy to reveal the pathogenic mechanisms and the host immune response although the RNA sequencing could only reflect the immune response of the pathogen or host. In this sense, the cytokine–cytokine receptor interaction pathway, two-component system, and bacterial secretion system are more likely to be involved in the immune response during *Salmonella* infection.

Evaluating host–SE interactions not only helps uncover the pathogenesis of SE infections, but also has the potential to develop to antimicrobial drug targeting. For example, sMtb-RECON was established by a combination model of bacteria *Mycobacterium tuberculosis* (Mtb) and human macrophage-like cell line THP-1 metabolic processes used as the elucidation of metabolic drug responses in a manner that has the potential to reduce antibiotic abuse (Rienksma et al., 2019). In another study, a dual RNA-sequencing time-course approach was used to monitor transcriptomic responses in both *Salmonella typhimurium* and infected HeLa cells, revealing a link between the *S. typhimurium* PinT virulence gene and the induction of immune signaling responses in HeLa cells via JAK-STAT signaling pathways (Westermann et al., 2016). In the present study, we find that *BPSS1504* was involved in SE infection, which served as a Type VI secretion system (T6SS) component capable of influencing hemolysin-coregulated protein Hcp1 secretion and T6SS apparatus integrity (Hopf et al., 2014). The interaction between *BPSS1504* and T6SS might be a potential target to develop an antimicrobial drug for SE clearance.

## Conclusion

In conclusion, the 10,510 euk-DEGs and 265 pro-DEGs were screened during duck SE challenge using dual RNA-seq, which enriched in the host cytokine–cytokine receptor interaction pathway and bacterial two-component/secretion system throughout the process, respectively. Also, a number of PHIs between cytokine–cytokine receptors and virulence-associated genes were found in this process based on WGCNA. Furthermore, the intracellular network associated with the regulation of SE infection in ducks was constructed, and 16 cytokine response–related dGC DEGs (including *IL15*, *CD40*, and *CCR7*, et al.) and 17 pathogenesis-related factors (including *sseL*, *ompR*, and *fliC*, et al.) were identified. Also, the interaction between *BPSS1504* and T6SS might be involved in immune regulation during SE infection. These data offer novel insights into the complex HPIs that occur upon SE infection of ducks, and unveil novel potential targets for SE infection diagnosis and antimicrobial drug development.

## Data Availability Statement

The datasets presented in this study can be found in online repositories. The names of the repository/repositories and accession number(s) can be found below: Short Read Archive (SRA), PRJNA721551.

## Ethics Statement

The animal study was reviewed and approved by The Institutional Animal Care and Use Committee (IACUC) of Yangzhou University (Jiangsu, China).

## Author Contributions

QX and GC conceived and designed the experiments. GZ assisted in experimental design. YZ and LS performed the experiments. LH, ZC, and WV analyzed the data. YZ wrote the manuscript. All authors contributed to the article and approved the submitted version.

## Conflict of Interest

The authors declare that the research was conducted in the absence of any commercial or financial relationships that could be construed as a potential conflict of interest.

## Publisher’s Note

All claims expressed in this article are solely those of the authors and do not necessarily represent those of their affiliated organizations, or those of the publisher, the editors and the reviewers. Any product that may be evaluated in this article, or claim that may be made by its manufacturer, is not guaranteed or endorsed by the publisher.

## Footnotes

1
https://github.com/najoshi/sickle

2
https://github.com/jstjohn/SeqPrep

3
http://broadinstitute.github.io/picard/

4
https://cran.r-project.org/web/packages/gmodels/index.html

## Supplementary Material

Supplementary Figure 1 Sample cluster map of dGCs and SE. **(A)** The results of dGCs. **(B)** The results of SE.

Supplementary Figure 2 Visualization of GO enrichment analysis of difference expression genes between 0, 3, 6, and 9 hpi during SE infection. All DEGs in specific-stage infection were analyzed by KEGG enrichment. Fold change > 2 and FDR < 0.01 were set as cutoff values. 0 vs. 3 hpi **(A)**, 3 vs. 6 hpi **(B)**, 6 vs. 9 hpi **(C)** of dGCs in different contrasts, respectively. 3 vs. 6 hpi **(D)**, 6 vs. 9 hpi **(E)**, 3 vs. 9 hpi **(F)** of SE in different contrasts, respectively.

Supplementary Figure 3 Visualization of gene expression levels and KEGG enrichment analysis of significant modules. Clustering heat map and bar plot represent gene expression levels of each module. In the heat maps, the colors range from green to red, indicating low to high expression levels, respectively. Panels **(A–D)** and panels **(E–H)** represent blue, dark red, black, and light cyan modules.

Supplementary Figure 4 dGC clustering of dDEGs (scaled and centered log2 values) over the time course of the infection. Gray lines represent the expression of individual genes; black lines are shown the variance; *p*-value < 0.05.

Supplementary Figure 5 SE clustering of DEGs (scaled and centered log2 values) over the time course of the infection. Gray lines represent the expression of individual genes; black lines are shown the variance; *p*-value < 0.05.

Supplementary Table 1 Intracellular CFU counts of SE at 0, 3, 6, and 9 h post-infection.

Supplementary Table 2 Summary of dual RNA sequencing for the samples.

Supplementary Table 3 Base information statistics before and after filtering.

Supplementary Table 4 Reads aligning to the SE and duck reference.

Supplementary Table 5 Analysis of dGCs differentially expressed mRNAs between 0, 3, 6, and 9 h post-infection.

Supplementary Table 6 Analysis of SE differentially expressed mRNAs between 3, 6, and 9 h post-infection.

Supplementary Table 7 GO enrichment analysis of dGCs difference expression genes between 0, 3, 6, and 9 h post-infection.

Supplementary Table 8 GO enrichment analysis of SE difference expression genes between 3, 6, and 9 h post-infection.

Supplementary Table 9 Identification of stage-specific modules based on weighted gene co-expression network analysis.
