# Supplementary figures and images for: Dual Transcriptomic Analyses Unveil Host–Pathogen Interactions Between Salmonella enterica Serovar Enteritidis and Laying Ducks (Anas platyrhynchos)
Source: Front Microbiol. 2021 Aug 5;12:705712. doi: 10.3389/fmicb.2021.705712 (PMC8374152; doi:10.3389/fmicb.2021.705712)

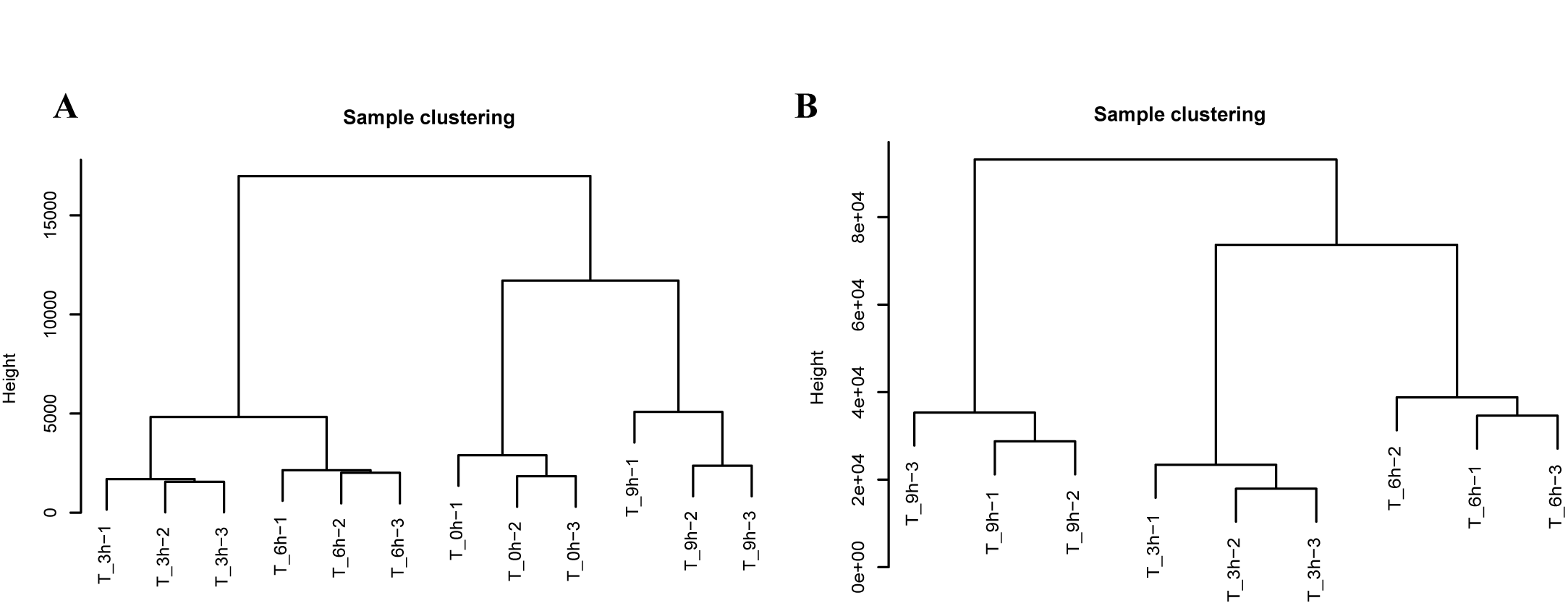

Supplement: Supplementary Figure 1 — Sample cluster map of dGCs and SE. (A) The results of dGCs. (B) The results of SE. [file Image_1.TIF]

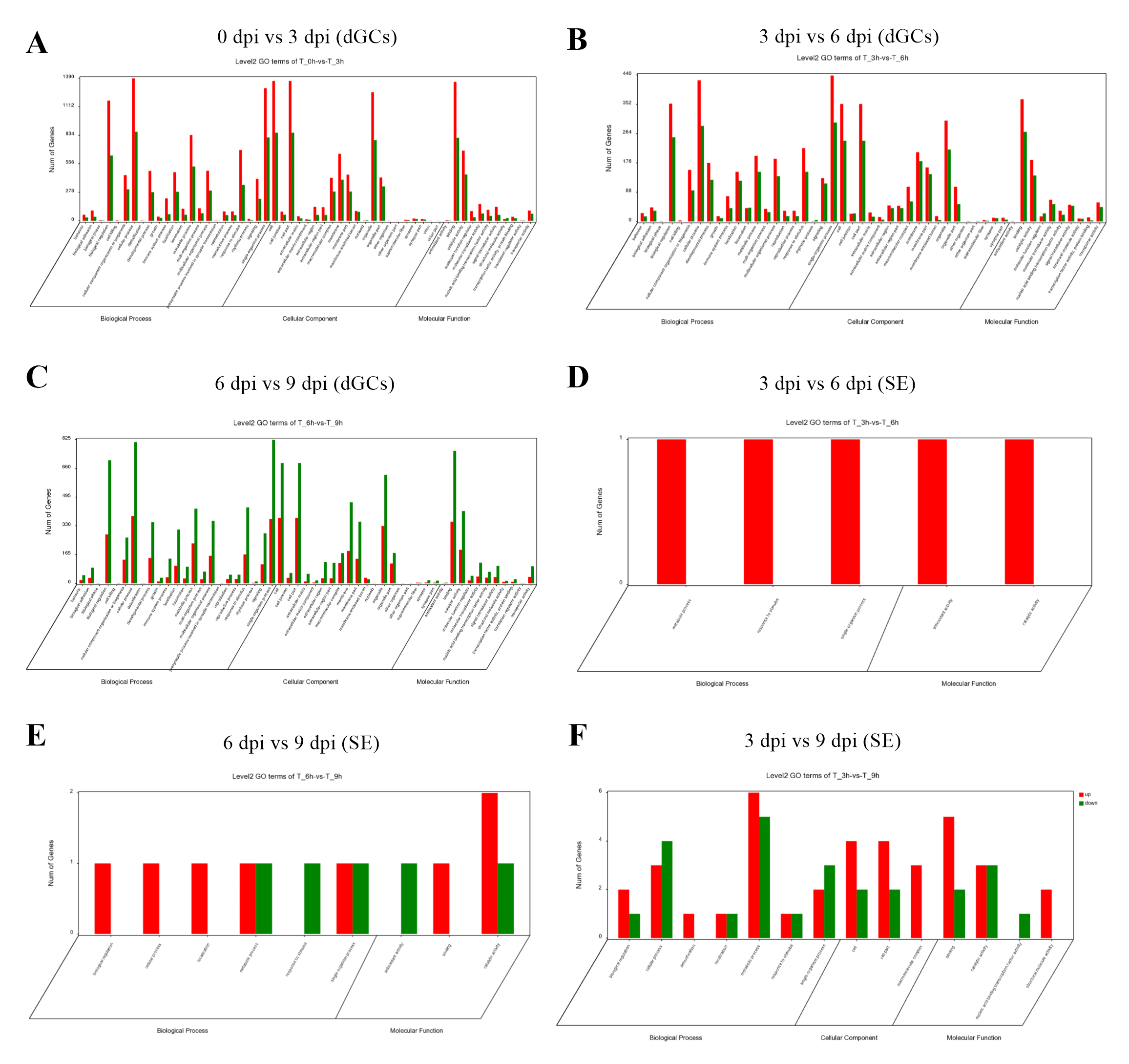

Supplement: Supplementary Figure 2 — Visualization of GO enrichment analysis of difference expression genes between 0, 3, 6, and 9 hpi during SE infection. All DEGs in specific-stage infection were analyzed by KEGG enrichment. Fold change > 2 and FDR < 0.01 were set as cutoff values. 0 vs. 3 hpi (A), 3 vs. 6 hpi (B), 6 vs. 9 hpi (C) of dGCs in different contrasts, respectively. 3 vs. 6 hpi (D), 6 vs. 9 hpi (E), 3 vs. 9 hpi (F) of SE in different contrasts, respectively. [file Image_2.TIF]

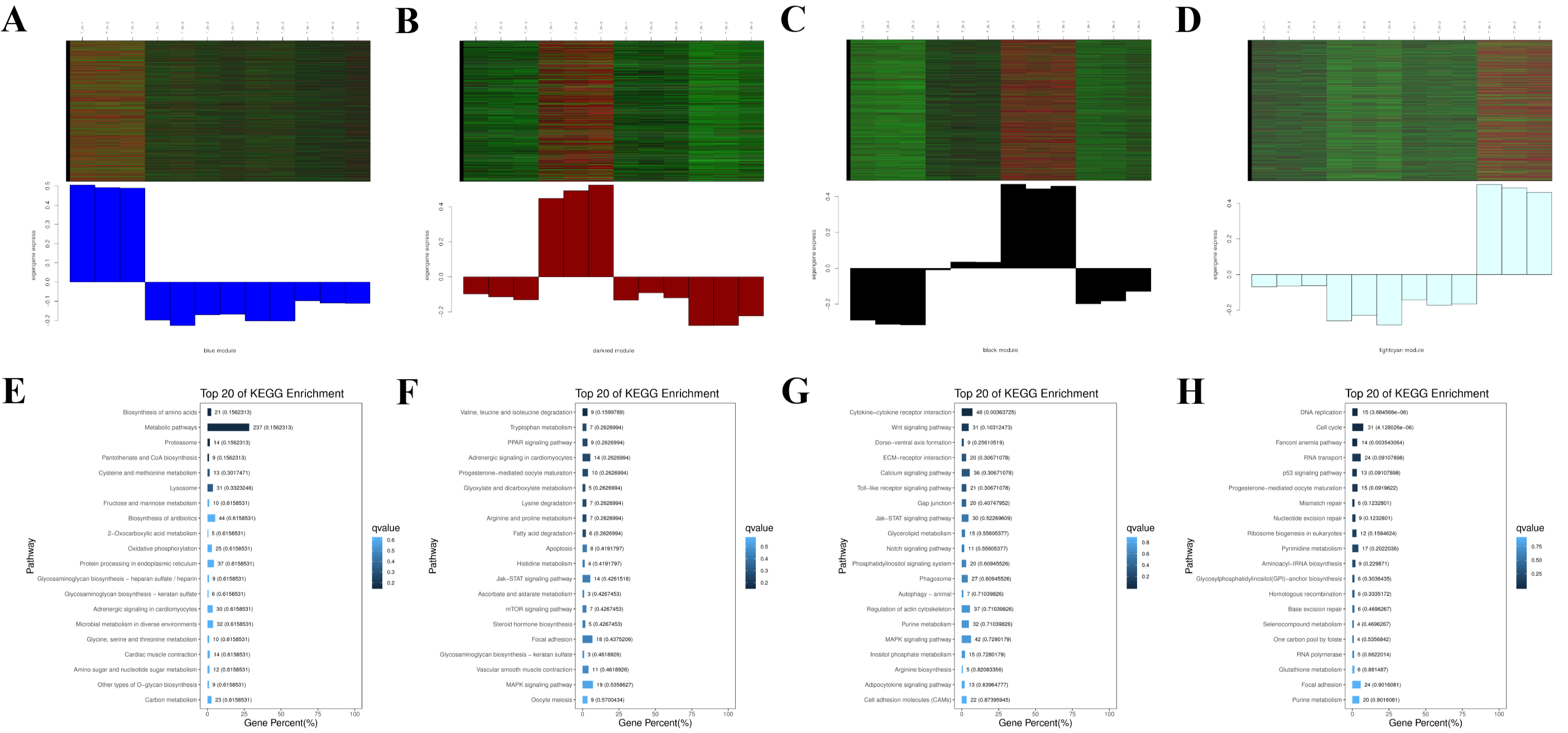

Supplement: Supplementary Figure 3 — Visualization of gene expression levels and KEGG enrichment analysis of significant modules. Clustering heat map and bar plot represent gene expression levels of each module. In the heat maps, the colors range from green to red, indicating low to high expression levels, respectively. Panels (A–D) and panels (E–H) represent blue, dark red, black, and light cyan modules. [file Image_3.TIF]

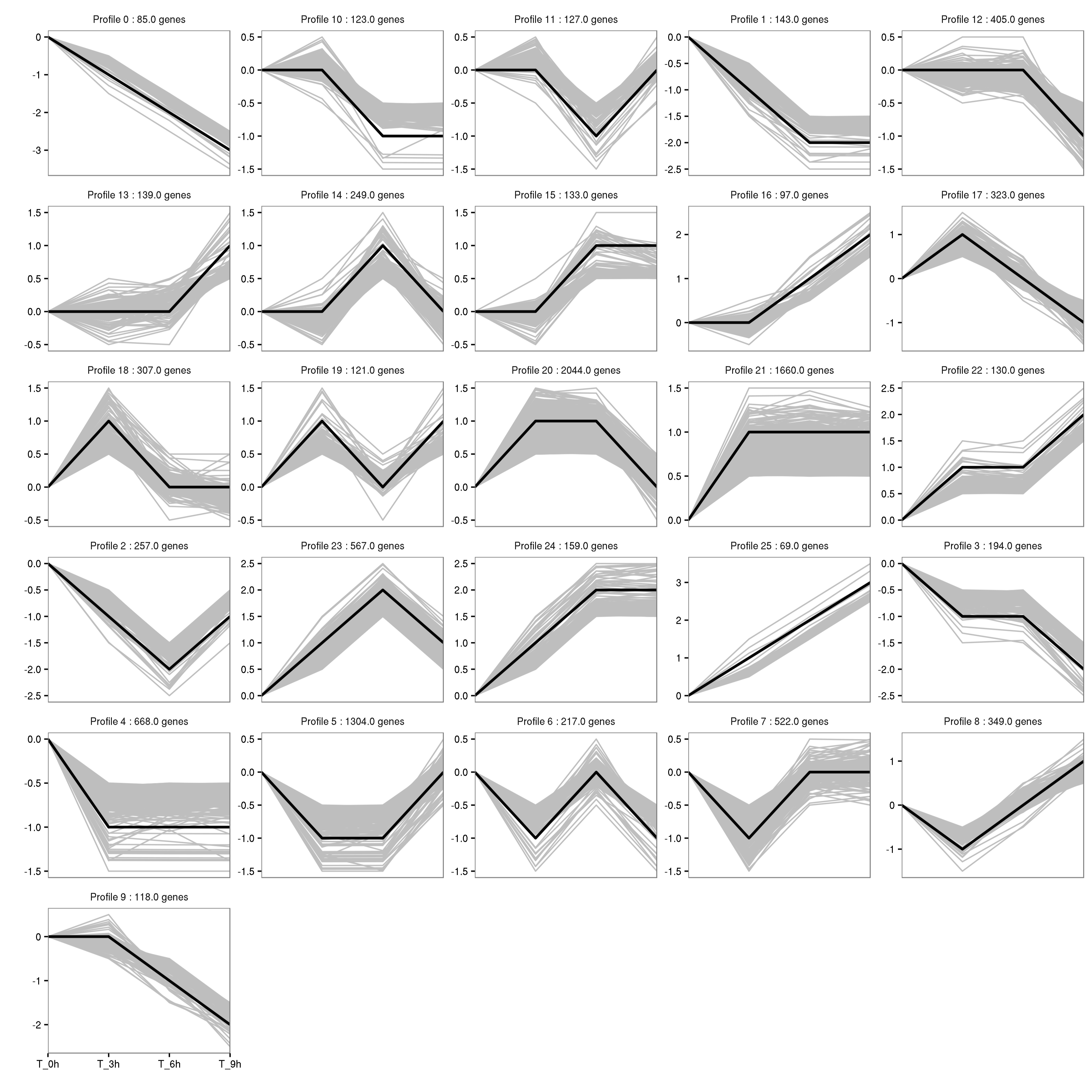

Supplement: Supplementary Figure 4 — dGC clustering of dDEGs (scaled and centered log2 values) over the time course of the infection. Gray lines represent the expression of individual genes; black lines are shown the variance; p-value < 0.05. [file Image_4.TIF]
